# WU Polyomavirus Infection in Children With Acute Lower Respiratory Tract Infections in China, 2017 to 2019: Case Reports and Multicentre Epidemiological Survey

**DOI:** 10.3389/fcimb.2021.835946

**Published:** 2022-03-11

**Authors:** Hongwei Zhao, Wenmiao Xu, Lijuan Wang, Yun Zhu, Xiaohui Wang, Yingchao Liu, Junhong Ai, Qianyu Feng, Li Deng, Yun Sun, Changchong Li, Rong Jin, Yunxiao Shang, Hengmiao Gao, Suyun Qian, Lili Xu, Zhengde Xie

**Affiliations:** ^1^ Beijing Key Laboratory of Paediatric Respiratory Infection Diseases, Key Laboratory of Major Diseases in Children, Ministry of Education, National Clinical Research Center for Respiratory Diseases, National Key Discipline of Paediatrics (Capital Medical University), Beijing Paediatric Research Institute, Beijing Children’s Hospital, Capital Medical University, National Center for Children’s Health, Beijing, China; ^2^ Research Unit of Critical Infection in Children, Chinese Academy of Medical Sciences, 2019RU016, Beijing, China; ^3^ Department of Paediatric Critical Care Medicine, Beijing Children’s Hospital, Capital Medical University, National Center for Children’s Health, Beijing, China; ^4^ Department of Respiration, Guangzhou Women and Children’s Medical Center, Guangzhou, China; ^5^ Department of Pediatrics, Yinchuan Women and Children Healthcare Hospital, Yinchuan, China; ^6^ Department of Pediatric Respiratory Medicine and Sleep Medicine, The Second Afliated Hospital and Yuying Children’s Hospital of Wenzhou Medical University, Wenzhou, China; ^7^ Department of Pediatrics, Guiyang Maternal and Child Health Hospital, Guiyang, China; ^8^ Department of Pediatric Respiratory, Shengjing Hospital of China Medical University, Shenyang, China

**Keywords:** WU polyomavirus, acute lower respiratory tract infection, fatal case, multicentre study, epidemiological survey

## Abstract

WU polyomavirus (WUPyV) is a novel member of the family *Polyomaviridae* recently detected in respiratory tract specimens. So far, it has not been proven whether WUPyV is a real causative agent for respiratory diseases. In this study, we described two patients with fatal infection who had WUPyV detected in their nasopharyngeal swabs. Furthermore, we conducted a multicentre study in six hospitals from different districts of China. WUPyV was detected by real-time polymerase chain reaction assays, and the clinical and molecular epidemiological characteristics of WUPyV strains among hospitalized children with acute lower respiratory tract infections all around China from 2017 to 2019 were analysed. Two complete WUPyV genome sequences were assembled from fatal patients’ airway specimens. Phylogenetic tree analysis revealed that they were most closely related to strains derived from Fujian and Chongqing, China, in 2008 and 2013, respectively. In 2017–2019, a total of 1,812 samples from children with acute lower respiratory tract infections were detected for WUPyV, of which 11 (0.6%) were positive. Children aged ≤5 were more susceptible to WUPyV infection. A total of 81.8% of WUPyV-positive patients were coinfected with other viruses, of which rhinovirus enjoyed the highest frequency. The main clinical symptoms of infected patients include fever, coughing and sputum expectoration. Most patients were diagnosed with pneumonia, followed by bronchial surgery. Three patients manifested severe infection, and all patients improved and were discharged. Our results show that WUPyV persistently circulates in China. Further investigations on the clinical role and pathogenicity of WUPyV are necessary.

## Introduction

Acute respiratory infection (ARI) has always been one of the important causes of hospitalization in children and has serious consequences, especially in young children under 5 years of age. Respiratory viruses are recognized as the leading cause of children with ARI and can be detected in more than 50% of cases (Burstein et al., 2019). In addition to traditional respiratory viruses, a number of new viruses have been detected in respiratory samples with the development of molecular biology detection technology. WU polyomavirus (WUPyV) was first discovered in 2007 and named after the laboratory it was found. It is an icosahedral, non-enveloped virus with a capsid composed of 72 pentameric capsomeres that surround a circular, double-stranded viral DNA genome of approximately 5.2 kb (Bialasiewicz et al., 2010).

Following the discovery of WUPyV in Australia, the virus was detected in specimens from patients with respiratory tract disease on all continents, suggesting a worldwide distribution (Gaynor et al., 2007). The detection rate in respiratory samples from children with respiratory disease varies from 0.35% to 16.4% (Babakir-Mina et al., 2013). The age of WUPyV-infected patients ranged from a few weeks to 53 years, and children <3 years of age dominated. Infections were predominantly detected in late winter, spring, and early summer (Zhuang et al., 2011). High infection rates were reported for study populations preselected for lack of immunocompetence (Sharp et al., 2009).

Although detected in respiratory specimens from ARI patients, it often appears with other pathogens. Whether WUPyV acts as a respiratory pathogen causing acute infection requires further discussion (Dinwiddie et al., 2016). The detection of WUPyV is not restricted to ARI patients; it could also be detected in stool samples (Babakir-Mina et al., 2009; Mourez et al., 2009), even in patients without any apparent clinical respiratory symptoms (Pena et al., 2019). However, it has to be determined whether WUPyV is able to replicate in the intestine or whether it accumulates by oral ingestion.

In this study, we detected the nucleic acid of WUPyV from nasopharyngeal swabs of two fatal cases with respiratory infection by metagenomic next-generation sequencing in 2020. Complete genome sequences were obtained for variations and phylogenetic analyses. Moreover, we conducted a multicentre observational study aimed to determine the prevalence of WUPyV infection among young children with ARI in different areas of China.

## Materials and Methods

### Patients and Clinical Samples

For two fatal cases, nasopharyngeal swabs were collected during acute respiratory infection episodes from the paediatric intensive care unit (PICU) of Beijing Children’s Hospital. Specimens were stored frozen (-80°C) until subjected to metagenomic analyses.

We performed a multicentre prospective study with children under the age of 18 years who were admitted to the hospital for acute lower respiratory tract infections from November 2017 to November 2019. Patients were enrolled from six cities, representing six different areas of China. The hospitals included Beijing Children’s Hospital in Beijing (central China), Yinchuan Maternal and Child Health Hospital in Yinchuan (northwestern China), Shengjing Hospital of China Medical University in Shenyang (northeastern China), The First Affiliated Hospital of Guangzhou Medical University in Guangzhou (southern China), Yuying Children’s Hospital of Wenzhou Medical University in Wenzhou (southeastern China), and Guiyang Women and Children Health care Hospital in Guiyang (southwestern China). Patient clinical records and information were anonymized and deidentified prior to analysis. Sputum specimens (from Wenzhou) or nasopharyngeal aspirates (NPAs, from the other five cities) were obtained during hospitalization and stored frozen (-80°C) until analysis. All patients were enrolled in a network that monitored viral pathogens of children with acute lower respiratory tract infections, and all the patients’ information was submitted to the Infectious Disease Surveillance System of China. This study was performed in strict accordance with the human subject protection guidance and approved by the Ethical Review Committee of Beijing Children’s Hospital. Written informed consent was obtained from the participants’ parents or guardians.

### Metagenomic Analyses

Nucleic acids were extracted from each nasopharyngeal swab sample with the Direct-zol RNA MiniPrep Kit (Zymo Research, Irvine, CA, USA) and TRIzol LS (Thermo Fisher Scientific, Carlsbad, CA, USA). The DNA/RNA concentrations were measured by a Qubit Fluorometer (Thermo Fisher Scientific). The sequencing library was constructed through transposase-mediated methods, and PCR amplification was performed (Life Technologies, Carlsbad, CA). The quality of the DNA libraries was assessed using a Qsep1 biofragment analyser (BiOptic, La Canada Flintridge, CA) to measure the adapters and the sizes of fragments before sequencing. Qualified DNA libraries were pooled together and sequenced on the NextSeq 550Dx sequencing platform (Illumina, San Diego, CA, USA). A single-end sequencing strategy was used, and the sequencing length was 75 bp. The primary sequencing output was demultiplexed by bcl2fastq v2.17.1.14. The reads were quality trimmed and subsequently filtered if shorter than 20 bases by Trimmomatic v0.32. Reads that passed these filters were aligned against human and synthetic (including synthetic normalization molecule control and sequencing adapter) references using Bowtie v2.2.4. Reads that aligned to either were set aside. Reads potentially representing human satellite DNA were also filtered *via* a k-mer-based method. The remaining reads were aligned with our microorganism reference database using BLAST v2.2.30. Reads with alignments that exhibited both high percent identity and high query coverage were retained, with the exception of reads that aligned with any mitochondrial or plasmid reference sequences. All tools were run with default parameters unless otherwise specified.

### Alignment and Phylogenetic Analysis

Multiple-sequence alignments were constructed with MAFT (https://www.ebi.ac.uk/Tools/msa/mafft/) software (version 7.407) using the accuracy-oriented method (L-INS-i). Phylogenetic trees were generated using the neighbour-joining method, and bootstrap values of MEGA software version 7 with 1,000 replicates were calculated to evaluate confidence estimates.

### PCR Detection of WUPyV From Specimens

Viral nucleic acids in the clinical samples were extracted using the NucliSens easyMAG system (bioMérieux, Marcy-l’Etoile, France) according to the manufacturer’s instructions. WUPyV was detected using real-time PCR with primers WU&KI-F 5′-CTTGCTGTRCCTGAAATWATWGC-3′, WU&KI-R5′-TAARCCTTCACCAGTWGCTATKGC-3′ and the MGB probe WU&KI-P FAM-AGCTGGAGGAGCAGAGGCMYTRTCAATTG-BHQ-2. Ct values less than 37 were considered positive. All the positive samples underwent a second PCR round specific for WUPyV, with primers AG0044 (5′-TGTTACAAATAGCTGCAGGTCAA-3′) and AG0045 (5′-GCTGCATAATGGGGAGTACC-3′) for the VP2 gene and primers AG0048 (50-TGTTTTTCAAGTATGTTGCATAA-30) and AG0049 (50-CACCCAAAAGACACTTAAAAGAAA-30) for the large T antigen (LTAg) gene (Lin et al., 2008). The PCR products were purified and sequenced using an ABI3730xl DNA Analyser at Sino Geno Max (Beijing, China).

### Coinfection Virus Detection

The Luminex xTAG respiratory viral panel (RVP) assay and Luminex 200 instrument (Luminex, Austin, TX) were used to detect 18 common respiratory viral pathogens and subtypes in the nucleic acid samples, including influenza A, influenza A subtype H1, influenza A subtype H3, 2009 H1N1, influenza B, human adenoviruses (HAdVs), human metapneumovirus (HMPV), human parainfluenza virus (HPIV) 1-4, respiratory syncytial virus (RSV) A and B, enteroviruses and rhinoviruses (EVs/RhVs), human coronavirus (HCoV) HKU1, 229E, NL63 and OC43, and human bocavirus (HBoV). Bacterial infection tests were not performed in our study.

## Results

### Fatal Case Presentations

#### Case 1 (BCH2008-1)

**Figure 1:**
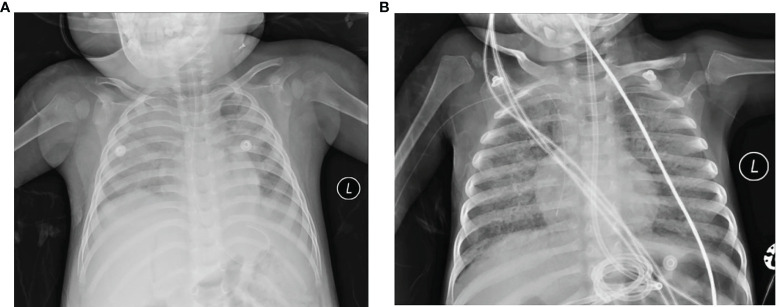
Chest X-ray of the lungs from the acute phage of the two fatal cases. **(A)** Case 1. Acute phage of pneumonia revealed the texture of the two lungs as thickened and fuzzy, the light transmittance of the left lung is diffusely reduced, left diaphragm blurred, the light transmittance of the right lung unevenly reduced, patchy shadows shown in the right lung. **(B)** Case 2. The lung light transmittance was unevenly reduced, and multiple high-density shadows and small air bubble emphysema were seen in both lungs. Part of the bronchiectasis and part of the cavity were irregular, especially in the middle lobe of the right lung.

A boy aged 1 year and 6 months was admitted to our hospital in March 2020 because of fever. He was full-term-born with no family genetic disease. Twenty days before admission, he was diagnosed with Epstein–Barr virus (EBV)-associated haematopoietic syndrome, and chronic active EBV infection chemotherapy was conducted regularly after diagnosis. Three days before admission, his body temperature reached 38.2°C, without convulsions, cough, expectoration, running nose, or vomiting, and his temperature returned to normal after taking an antipyretic. Laboratory examination data showed decreased red blood cell and white blood cell counts but no abnormalities in PCT and CRP. Chest computed tomography (CT) revealed mild consolidation and a small amount of fluid in the left pleural cavity (**Figure 1A**). Other laboratory results are shown in **Table 1**. Voriconazole, methylprednisolone, and luctinib were given immediately after his admission to our hospital. The day after admission, molecular detection of throat swabs was positive for *Mycoplasma*, and azithromycin was given intravenously. Six days later, the patient’s condition worsened, with weak bowel sounds and weak mental response, and the patient was transferred to the PICU on flat car under nasal cannula oxygen inhalation. Nasopharyngeal swab was collected the day after his transfer. Nasal continuous positive airway pressure (NCPAP) auxiliary breathing was given 6 h after his transfer. On Day 11, his transcutaneous oxygen saturation dropped drastically, his heart rate increased to approximately 180–200 per minute, and his blood pressure intermittently dropped to 70/40 mmHg. After cardiopulmonary resuscitation for 30 min, the patient died because of multiple organ dysfunction caused by haematopoietic syndrome and multiple inflammations.

**Table 1:** Laboratory detection and mNGS results of the two fatal cases.

	Case 1	Case 2
Red blood cell count (10^9^/L)	4.34	3.74
White blood cell count (10^9^/L)	2.08	1.82
Differential count (%)		
Neutrophils	1.78 (85.5%)	1.35 (74.3%)
Lymphocytes	0.18 (8.7%)	0.09 (4.9%)
Monocytes	0.11 (5.3%)	0.33 (18.1%)
Eosinophils	0 (0%)	0.04 (2.2%)
Basophils	0.01 (0.5%)	0.01 (0.5%)
Platelet count(10^9^/L)	250	289
Carbon dioxide pressure	42	46
mNGS results*^a^* (reads)		
Bacteria	*Enterococcus faecium* (9726)	None
Fungi	None	None
Virus	**WU Polyomavirus (28883)**	**WU Polyomavirus (17809)**
	Human gamma herpes virus 4 (2789)	Human bocavirus (387)
	Human beta herpes virus 5 (335)	Rubella virus (6338)

^a^Only pathogens with reads>300 were recorded.

#### Case 2 (BCH2020_1)

A 3-year-and-8-month-old boy who had primary combined immunodeficiency [X-linked severe combined immunodeficiency in the 1L2RG gene (OMIM: 312863), and X-linked severe combined immunodeficiency highly associated mutation (OMIM: 300400)] and experienced interstitial pneumonia at 2 years of age was admitted to our hospital for fever, cough and difficulty breathing. He was also a full-term child, and there was no abnormality in his birth history. He had been admitted to local hospitals 11 times since his first admission for “interstitial pneumonia” in 2019. He was transferred to the PICU of our hospital with a diagnosis of severe pneumonia, respiratory failure and interstitial pneumonia. Nasopharyngeal swabs were collected the next day. Coarse breath sounds and moist rates were found in both lungs. Chest CT demonstrated diffuse parenchymal changes and small alveolar emphysema in both lungs (**Figure 1B**). Sputum specimen was positive for *Pneumocystis*. Other clinical results are shown in **Table 1**. Upon admission, cotrimoxazole and methylprednisolone were given immediately to control infection and relieve inflammation. Fourteen days later, fungal hyphae were found in the respiratory tract, and voriconazole was added. On Day 87, his dyspnoea increased, and mechanical ventilation was given. On the 98th day of admission, his transcutaneous oxygen saturation, heart rate, and blood pressure dropped severely, with spontaneous breathing and aorta pulsates disappearing. He finally died due to severe acute respiratory distress syndrome that developed from pneumonia.

### Viral Sequence Analyses from Two Fatal Cases

Two complete genome sequences of 5,229 bp (BCH2008-1) and 5,228 bp (BCH2020_1) were identified after mapping 35,550 (29,374,647 reads in total; 0.12%) and 56,897 (37,864,592 reads in total; 0.15%) sequencing reads, respectively, against the 5,229-bp-long reference genome for WUPyV (GenBank accession no. NC_009539). The whole-genome sequences of two WUPyV strains have been deposited in GenBank under accession numbers MW338654 and MW338655 (Zhao et al., 2021). The alignment results of the two sequences with the reference sequence are shown in **Table 2**. For BCH2008-1, there was only one amino acid variant in the VP2 protein (Glu250Gly) alignment to NC_009539. For BCH2020_1, there were three variants separately located in VP1 (Ala82Thr), VP2 (Met324Ile), and large T antigen (Asn56Lys). All four variations were not unique for our cases. After summarizing all WUPyV complete sequences submitted to GenBank (149 in total), the incidence of variations found in our sequences is shown in **Table 2**.

**Table 2:** Non-synonymous changes in the amino acids of two WUPyV strains from fatal cases when aligned with the reference strain (GenBank accession no. NC_009539) and the incidences of each variation in all 149 complete WUPyV genomes submitted to GenBank.

	Amino acid mutation	Encoded protein region	Incidence (%, total no. = 149)
MW338654.2 (BCH2008_1)	Glu250Gln	VP2/3	44.3%
MW338655.2 (BCH2020-1)	Ala82Thr	VP1	29.5%
	Met324Ile	VP2/3	8.7%
	Ile594Leu	Large T antigen	70.5%

**Figure 2:**
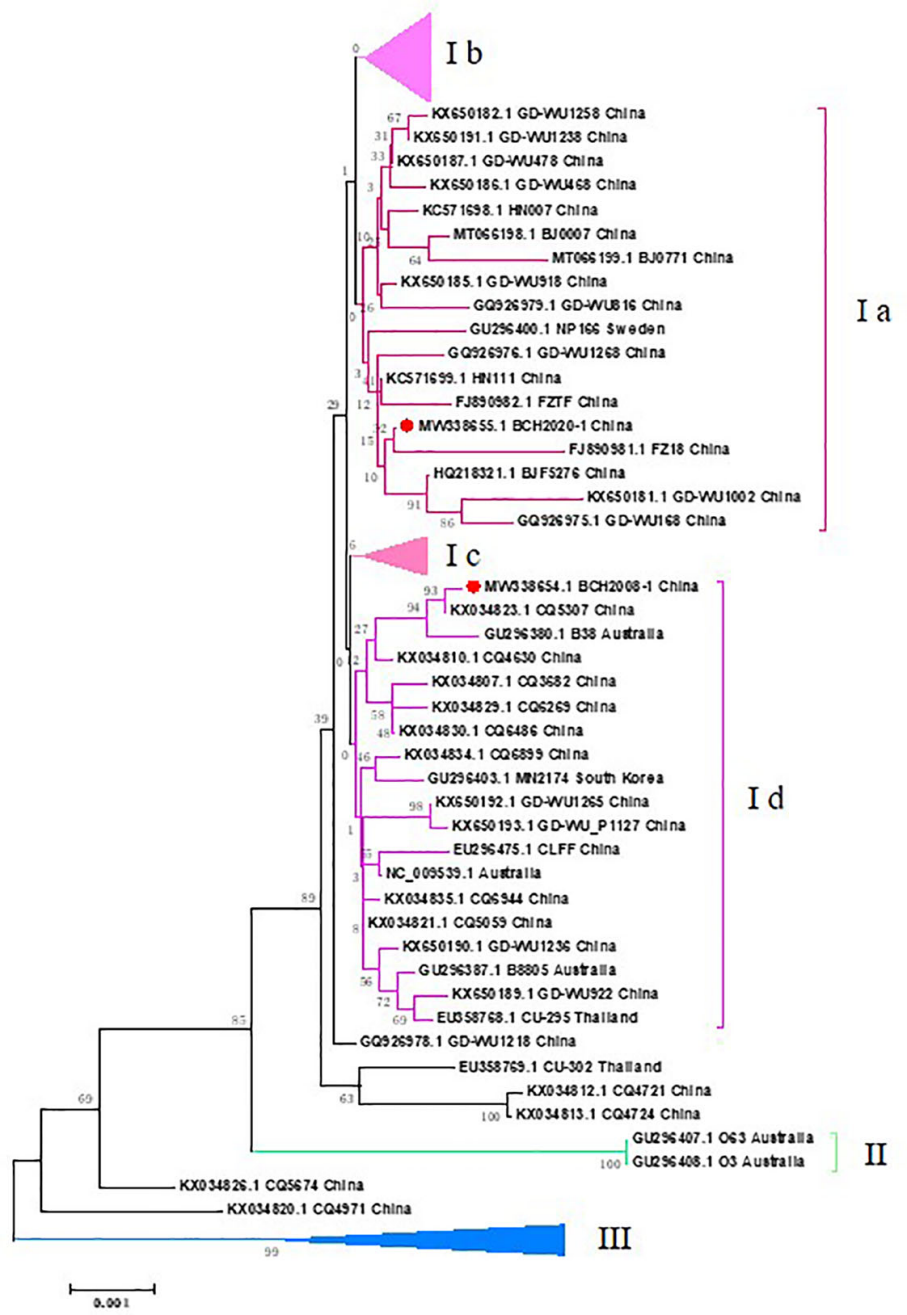
Phylogenetic tree based on the two WUPyV complete genome sequences from fatal cases. Phylogenetic analyses were performed with other 114 WUPyV complete sequences submitted to GenBank, with all sequences derived from China (67 in total) and representative sequences from other countries (23 from Australia, 2 from Thailand, 5 from Germany, 3 from Netherlands, 5 from Canada, 3 from Sweden, 3 from South Korea, and 1 from the USA). MEGA v7.0 software was used to generate phylogenetic trees with the neighbor-joining method and the Kimura 2-parameter model. The robustness of the phylogenetic trees was assessed using the bootstrap method with 1,000 replicates. Reference genomes with bootstrap values below 70 are not shown. The strains in the current study are marked with a solid red circle.

Phylogenetic analyses were performed with 112 other WUPyV complete sequences submitted to GenBank, with all sequences derived from China (67 in total) and representative sequences from other countries (23 from Australia, 2 from Thailand, 5 from Germany, 3 from the Netherlands, 5 from Canada, 3 from Sweden, 3 from South Korea, and 1 from the USA). The results showed that three branches were formed, and the largest branch (I) was further divided into four clusters. The strains in Cluster Ib were mainly from Western countries, and strains from China were gathered in Cluster Ia, Cluster Ic, and Cluster IIIa. Our isolate BCH2020-1 grouped most closely with the strain in Cluster Ia: FZ18 (accession no. FJ890981.1), which was collected in 2013 from Fujian, China. BCH2008_1 grouped most closely with strain CQ5307 (accession no. KX034823.1), which was collected from Chongqing, China, in 2013 (**Figure 2**).

### Epidemiological Analyses

A total of 1,812 respiratory specimens were collected from six hospitals between January 2017 and December 2019, of which 544 were collected in Beijing, 279 in Shenyang, 277 in Wenzhou, 307 in Yinchuan, 241 in Guangzhou, and 317 from Guiyang. A total of 439 specimens were collected in 2017, 784 were collected in 2018, and 589 were collected in 2019 (**Table 3**). 770 (42.5%) patients were female.

**Table 3:** Specimen collection time and regions of the multicentre study.

Region	City	Number of samples	Year		
			2017 (%)	2018 (%)	2019 (%)
North	Beijing	544	158 (29.0%)	236 (43.4%)	150 (27.6%)
	Yinchuan	279	120 (43.0%)	80 (28.7%)	79 (28.3%)
	Shenyang	174	38 (21.8%)	136 (78.2%)	0
South	Wenzhou	272	10 (5.6%)	180 (66.2%)	82 (29.4%)
	Guiyang	317	39 (12.3%)	152 (47.9%)	126 (39.7%)
	Guangzhou	241	74 (30.7%)	0	167 (69.3%)
Total		1812	439 (24.2%)	784 (43.3%)	589 (32.5%)

By using universal PCR primers for WUPyV and KIPyV, 47 specimens were positive. However, further amplification with WUPyV-specific primers targeting VP2 and the LTAg genes verified that only 11 (5.6%) specimens were WUPyV positive. The clinical information of one positive patient was incomplete, and the other 10 patients’ demographic and clinical data are shown in **Table 4**.

**Table 4:** The demographic and clinical characteristics of patients positive for WUPyV.

	Cases (%, n = 10*^a^*)
Age (months)	
Median	27
Range	4-44
Sex	
Male	6 (60%)
Female	4 (40%)
Underlying diseases	2 (20%)
Symptoms and signs	
Cough	9 (90%)
Rhinorrhea	2 (20%)
Vomiting	1 (10%)
Diarrhoea	2 (20%)
Phlegm	4 (40%)
Fever	7 (70%)
Radiographic abnormality	8 (80%)
Clinical diagnosis	
Pneumonia	7 (70%)
Bronchiolitis	3 (30%)
Upper respiratory infection	1 (10%)

^a^One patient’s information missed.

Of these patients, four were from Beijing (positive rate was 0.7% for the district, the following was the same), three were from Wenzhou (1.1%), two were from Guangzhou (0.8%), one was from Guiyang (0.3%), and one was from Yinchuan (0.3%). Five specimens were collected in the winter season (October to February), five in spring (March to May), and only one in August. Their median age was 40 months (range from 4 months to 11 years), and eight patients were under 5 years of age. Six (54.5%) of them were boys. Two patients had medical histories: one suffered from acute granulocytic leukaemia, and another was diagnosed with bronchial asthma, which may increase the possibility of infection in the respiratory tract.

In this study, patients with WUPyV infection often showed fever, cough and sputum expectoration, and some patients may have gastrointestinal symptoms, such as diarrhoea. Chest radiographs were performed in 10 patients, nine of whom revealed signs of pneumonia. The most common diagnosis was pneumonia (7, 63.6%), followed by bronchitis (3, 27.2%) and upper respiratory infections (1, 9.1%). There were no fatal cases in this epidemiological cohort study. Eight patients showed mild clinical symptoms. Three patients were diagnosed with severe pneumonia, and one was admitted to the intensive care unit. However, all these patients improved and were discharged (**Table 5**).

**Table 5:** More demographic and clinical data of the 11 patients who were positive for WUPyV.

District	Sample collection date	Sample type	Age	Sex	Coinfection pathogen	Severity of illness
Beijing	2017/1	NP	9y	F	MP	Mild
Beijing	2017/12	NP	7y	F	–	Mild
Beijing	2018/2	NP	11y	M	–	Severe
Beijing	2018/12	NP	3y4 m	M	EV/Rh	Mild
Wenzhou	2018/1	Sputum	8 m	M	ADV	Mild
Wenzhou	2018/4	Sputum	4m3d	M	RSV	Mild
Wenzhou	2018/5	Sputum	1y6 m	M	ADV, HBoV	Severe
Guangzhou	2018/4	NP	4y	F	EV/Rh, ADV, HBoV	Mild
Guangzhou	2019/8	NP	2y5 m	F	HBoV, PIV 3	Mild
Guiyang	2018/4	NP	2y7 m	M	HRV	Severe
Yinchuan	2018/4	NP	4 m	F	RSV	Mild

### Coinfection With Other Respiratory Viruses

Among 11 WUPyV-positive patients, nine (81.8%) were coinfected with other respiratory viruses (**Table 5**). Four patients were infected with only one virus, another four were coinfected with two viruses, and one was coinfected with 3 viruses. Coinfection with human bocavirus (HBoV) and adenovirus (AdV) was most common (3 each, 27.3%).

By using the Luminex xTAG 18 respiratory viral panel, two patients were detected as only infected with WUPyV. However, both of them showed elevated white blood cell counts, mainly neutrophils, with elevated CRP levels. Bacterial infection tests were not performed in our study, so we could not deduce whether clinical infection expression was induced by WUPyV only.

## Discussion

WUPyV is a novel member of the family *Polyomaviridae* recently detected in respiratory tract specimens (Gaynor et al., 2007). Some studies report the detection of WUPyV in stool or serum (Babakir-Mina et al., 2009). It can be found in ARI paediatric patients’ respiratory samples, especially in immunocompromised individuals, and sometimes causes severe infection. WUPyV infections cannot be distinguished from other viral infections by means of clinical symptoms. Respiratory tract diseases such as pneumonia or bronchitis are frequently observed in patients harbouring WUPyV. Fatal cases with WUPyV infection were sporadically reported recently (Uda et al., 2018); however, almost all the reported patients suffered from immunodeficiency disease.

Here, we detected WUPyV by mNGS with high reads in two patients with respiratory infection, indicating that active replication existed in the airways of two patients. However, other pathogens were also found with high reads. It seems that WUPyV was not the causative agent that caused the two children’s deaths. It is worth noting that our multicentre epidemiological study of acute lower respiratory tract infections showed that two patients were infected only by WUPyV, and none were infected by other common respiratory viruses. One patient even developed severe pneumonia. The lack of bacterial and fungal detection results was a barrier to the conclusion of whether WUPyV could act as a single pathogen for respiratory infection. To date, it has not been proven whether WUPyV is a real causative agent for respiratory diseases. It seems that WUPyV may share some similarity with other members of the *Polyomaviridae* family: infecting humans in a very early stage of life; acting as an opportunistic pathogen after the acute period; establishing a latent infection with subsequent symptomatic or asymptomatic reactivation; causing respiratory symptoms when the body immunity system is compromised; and leading to fatal infections (Shoji et al., 2021).

Previous studies showed that the genome of WUPyV was highly conserved with minimal variations, but phylogenetic analyses demonstrated a high genetic variety of WUPyV strains circulating in China (Zhu et al., 2017). There have been some suggestions that sequence variation plays a role in disease severity and pathogenesis in other polyomaviruses (Prado et al., 2018). In the two fatal cases, one and three variations were found in WUPyV strains. Alignment results showed that these variations also existed in other WUPyV strains worldwide. The relationship between viral genome variations and disease severity remains unclear. The severity of infection may be associated with patients’ immune status.

In our multicentre study, the positive rate of WUPyV was 0.6%. Previous detection rates in other counties and regions vary from 0.4% to 11.5%. It has been reported that higher detection rates of polyomavirus were observed among participants aged 0 to 10 years and among those aged 61 to 70 years, which may be explained by the lower immune response of these individuals (Pena et al., 2019). Rockett et al. showed that WUPyV was more commonly detected in the respiratory tract of healthy children <18 months of age and associated with mild upper respiratory symptoms (Rockett et al., 2015). Although the positive rate was much lower than that of common respiratory viruses in ARI patients, a serological epidemiological survey demonstrated high sustained rates of infection by WUPyV in individuals. In healthy adults, the prevalence of antibodies against WUPyV VP2 is up to 90% (Nguyen et al., 2009). Whether WUPyV is an opportunistic pathogen with pathogenic potential in the respiratory tract under conditions remains to be defined.

In conclusion, two fatal paediatric patients with WUPyV infections were reported. A multicentre study revealed that the positive rate for WUPyV was 0.6% from 2017 to 2019. Infection was more common in children under 5 years old and manifested with fever and respiratory infection symptoms. High rates of coinfection were observed.

## Data Availability Statement

The datasets presented in this study can be found in online repositories. The names of the repository/repositories and accession number(s) can be found in the article/supplementary material.

## Ethics Statement

The studies involving human participants were reviewed and approved by the Ethical Review Committee of Beijing Children’s Hospital. Written informed consent to participate in this study was provided by the participants’ legal guardian/next of kin. Written informed consent was obtained from the minor(s)’ legal guardian/next of kin for the publication of any potentially identifiable images or data included in this article.

## Author Contributions

HWZ performed the experiments and drafted the manuscript. WMX, LJW, XHW, and YCL handled the two fatal clinical cases. YZ, JHA, and QYF performed the data analysis. LD, YS, CCL, RJ, and YXS collected and provided multi-centre clinical specimens and information. LLX, ZDX, SYQ, and HG participated in the study design and coordinated the drafting of the manuscript. All authors contributed to the article and approved the submitted version.

## Funding

This work was funded by the National Natural Science Foundation of China (82172275) and the CAMS Innovation Fund for Medical Sciences (CIFMS, 2019-I2 M-5-026).

## Conflict of Interest

The authors declare that the research was conducted in the absence of any commercial or financial relationships that could be construed as a potential conflict of interest.

## Publisher’s Note

All claims expressed in this article are solely those of the authors and do not necessarily represent those of their affiliated organizations, or those of the publisher, the editors and the reviewers. Any product that may be evaluated in this article, or claim that may be made by its manufacturer, is not guaranteed or endorsed by the publisher.
